# Primary Oro-Facial Manifestations of Langerhans Cell Histiocytosis in Pediatric Age: A Bi-Institutional Retrospective Study on 45 Cases

**DOI:** 10.3390/children7090104

**Published:** 2020-08-19

**Authors:** Saverio Capodiferro, Angela Tempesta, Luisa Limongelli, Giuseppe Ingravallo, Eugenio Maiorano, Gian Luca Sfasciotti, Maurizio Bossù, Antonella Polimeni, Gianfranco Favia

**Affiliations:** 1 Department of Interdisciplinary Medicine, Complex Operating Unit of Odontostomatology, University of Bari “Aldo Moro”, Piazza G. Cesare, 11, 70124 Bari, Italy; angelatempesta1989@gmail.com (A.T.); luisanna.limongelli@gmail.com (L.L.); gianfranco.favia@uniba.it (G.F.); 2 Department of Emergency and Organ Transplantation, Operating Unit of Pathological Anatomy, University of Bari “Aldo Moro”, Piazza G. Cesare, 11, 70124 Bari, Italy; giuseppe.ingravallo@uniba.it (G.I.); eugenio.maiorano@uniba.it (E.M.); 3 Department of Oral and Maxillofacial Sciences, Unit of Paediatric Dentistry, “Sapienza” University, Via Caserta, 6, 00161 Rome, Italy; gianluca.sfasciotti@uniroma1.it (G.L.S.); maurizio.bossu@uniroma1.it (M.B.); antonella.polimeni@uniroma1.it (A.P.)

**Keywords:** Langerhans Cell Histiocytosis, pediatric age, oral manifestations, head and neck manifestations, early diagnosis

## Abstract

Aims: Langerhans Cell Histiocytosis is a rare hematologic disorder usually affecting children and most commonly involving the head and neck region. Primary oro-facial manifestations are rare, and their diagnosis is often challenging as they are numerous and often resemble common pathologies, refractory to conventional medical and/or instrumental treatments. For such reasons, the diagnosis is frequently delayed, as is the following staging and therapy onset. We retrospectively studied 45 pediatric patients affected by Langerhans Cell Histiocytosis with onset in the head and neck, to examine their clinical and radiological features at the early stage. Materials and Methods: The study was a retrospective bi-institutional analysis (Department of Pediatric Dentistry and Pediatric Oncology of “Sapienza” University of Rome, Department of Interdisciplinary Medicine of the University of Bari “Aldo Moro”), which enrolled 45 patients (age range 0–18 year-old) affected by Langerhans Cell Histiocytosis with oro-facial onset. Data regarding clinical appearance, number, site, synchronous or metachronous occurrence, involved tissues/organs, radiographic features and clinical outcomes were collected, listed and overall differentiated by two age ranges (0–10-year-olds and 10–18-year-olds). Results: Patients were 26 males and 19 females, with an average age at the time of diagnosis of 4.8 ± 3.8 years (median = 3.9 years). The most common findings were inflamed, hyperplastic, painful and often ulcerated gingival lesions (22 cases), associated with deciduous tooth mobility and/or dislocation with bone loss in 18 cases, followed by nine single eosinophilic granulomas of the mandible and two of the maxilla. Lesions of the palatal mucosa were observed in six patients; nine patients showed on radiograms the characteristic “floating teeth” appearance in the mandible with synchronous lesions of the maxilla in six. Paresthesia was relatively un-frequent (three cases) and the pathological fracture of the mandible occurred in six. Head/neck lymph nodes involvement was associated with oral lesions in 12 cases and skull lesions in 14. Otitis (media or externa) was detected in four instances, exophthalmia in two, cutaneous rush in nine, contextual presence or subsequent onset of insipidus diabetes in eight. As for therapy, single or multiple small jaw lesions were all surgically removed; chemotherapy with vinblastine alone or associated with corticosteroids was the principal treatment in almost the 80% of cases; more than 50% of patients received corticosteroids, while only three patients received adjunctive radiotherapy. The overall mortality account for less than 9% (four of 45 cases) and recurrence observed in eight patients after therapy. Conclusions: Langerhans Cell Histiocytosis may mimic several oro-facial inflammatory and neoplastic diseases. Considering the potential disabling sequela following head and neck localization of Langerhans Cell Histiocytosis in children, especially at the periodontal tissues with teeth and alveolar bone loss, lesion recognition along with the histological examination of suspicious tissues is mandatory to achieve an early diagnosis and to prevent further organ involvement.

## 1. Introduction

Langerhans Cell Histiocytosis (LCH), previously known as Histiocytosis X, is a rare hematologic disorder usually affecting children [1,2,3]. LCH is characterized by an abnormal proliferation of bone-marrow derived histocytes (dendritic cells resembling Langerhans cells of the epidermis) that may infiltrate and consequently damage single or multiple organs and tissues, especially the bones [1,4,5]. For such reasons, despite the conventional classification in three distinct clinical entities such as Eosinophilic Granuloma, Letterer–Siwe disease and Hand–Schüller–Christian disease, LCH is currently classified according to the treatment recommendations of the Histiocyte Society into Single-System LCH (SS-LCH) and Multi-System LCH (MS-LCH) [6]. More precisely, the eosinophilic granuloma, being an unifocal disease, is considered an SS-LCH as involving a single site; the Hand–Schüller–Christian disease is a multifocal SS-LCH because it occurs in multiple sites of a single organ system, while the Letterer–Siwe disease is a multifocal MS-LCH as it involves multiple sites in more than one organ. MS-LCH is further classified based on the presence or absence of a Risk Organ (RO) (e.g., central nervous system, liver, hematopoietic system, etc.) into RO^+^/RO^−^ [6].

LCH signs and symptoms can greatly differ according to the unifocal or multifocal clinical occurrence [2,7,8]. LCH affects fewer than five people per million with a rate of 0.2 to 0.5 cases per 100,000 children per year, with a peak of incidence between 3–4 years of age and with a male predilection of 1.6–1.7 increased occurrence than female [2,3,7,8]. LCH predominantly involves the skeleton, followed by the skin and the pituitary gland, while organs/systems commonly related to a higher risk of severe progression, such as bone marrow, liver, spleen and lungs, are less frequently involved.

Notably, 55% to 80% of cases of disseminated LCH may manifest lesions in the head and neck, especially of the skull (mostly of the temporal bone), lymph nodes, skin and orbit, while only 7–10% have oral lesions (mostly jaw lesions); moreover, primary manifestations in the oral cavity are rare [2,3,4,9,10,11]. Clinically, localized or multiple gingival swellings with edema/inflammation or ulceration (rarely), periodontal involvement with teeth mobility/dislocation, often associated to destroying bone lesions (especially of the posterior mandible) with the conventional appearance of “floating teeth” or “floating in air” teeth on radiograms, are common primary findings in not yet diagnosed cases [2,3,7,12]. Even less frequent is the primary onset of a single bone lesion (especially in the maxilla) with periosteal reaction, punch-out radiolucent appearance, absence of cortical bone or diffuse periodontal involvement, mimicking on radiograms aggressive and diffuse periodontal disease, as well as mucosal infiltration (almost exclusively of the palate) with slight proliferation, erythema and strawberry appearance [2,3,7,13,14].

In the current study, we describe retrospectively the primary oral and maxillo-facial lesions that led to the diagnosis of LCH in a cohort of 45 pediatric patients. With this clinical–pathological case series, we would like to emphasize the importance of general dentist’s knowledge of the precocious oral signs of systemic/generalized diseases, such as LCH, to recognize suspicious lesions at early stage and to rapidly set on the more fit treatment and finally improve both prognosis and quality of life in such patients.

## 2. Methods

The current retrospective bi-institutional study was carried out by the Departments of Pediatric Dentistry and Pediatric Oncology of “Sapienza” University of Rome, and Interdisciplinary Medicine of University of Bari “Aldo Moro”. From each database of pediatric patients affected by LCH with oro–maxillo-facial involvement in the last 3 decades, 45 cases were enrolled in the current study as showing primary head and neck lesions. Age of occurrence at the time of diagnosis was between 0 and 18 years. Lesions were detected by clinical and radiographic investigations (skull X-ray dental panoramic radiograph, skull CT, head–neck MRI and bone scintigraphy with technetium, accordingly to localization) and always confirmed by histological analysis of bioptic tissue. Overall, dento/oro/facial manifestations were carefully classified according to the clinical appearance, number, site, synchronous or metachronous occurrence, involved tissues/organs and radiographic features. Nevertheless, expecting a great variability of clinical appearance as well as the metachronous occurrence of multiple similar or different lesions with the consequent impossibility of cataloging them all separately, the authors decided to create three levels (A, B, C) of association frequency between clinical signs and disease, as follows: A = range 70–100%; B = range 40–70%; C = range 0–40%. Additionally, such association was separately distinguished by age ranges 0–10 and 10–18. All resulting data are listed in Table 1.

Paraffin blocks of all specimens were retrieved, cut and stained with Hematoxylin-Eosin, PAS and Toluidine blue; as for immunohistochemistry, we used a panel including CD1a, CD3, CD4, CD20, CD34, CD68, CD163, CD207 (Langerin) and S100. Factor XIIIa, CD163, Fascin and CD14 antigens were used as the control panel, being specific for other non-Langerhans Cell histiocytic disorders (juvenile xanthogranuloma and hemophagocytic lymphohistiocytosis), as listed in Table 2. Histological examination was performed by conventional optical microscopy.

This retrospective study was performed in accordance with the principles of Declaration of Helsinki and has been approved by our institution’s ethical committees (Study n° 3303, Prot. 869/14 for the University of Rome, and Study n° 4599, Prot. 1528/C.E. for the University of Bari “Aldo Moro”). All patients released informed consent at the time of hospitalization on diagnostic and therapeutic procedures and possible use of biologic samples for research purposes.

## 3. Results

Patients were 26 males and 19 females, with an average age at the time of diagnosis of 4.8 ± 3.8 years (median = 3.9 years). All patients presented at least one oral sign/symptom unresponsive to conventional medical therapies performed by the general dentist or were referred because of the occasional finding of clinical or radiological lesions of dubious interpretation at the pediatrician consultation.

**Figure 1:**
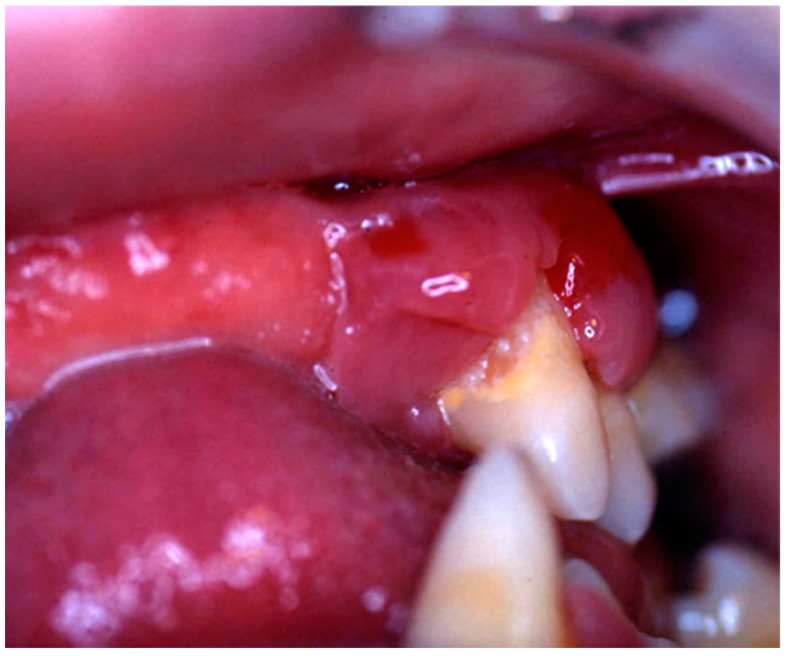
Hyperplastic, reddish and inflamed gingiva unresponsive to conventional medical and instrumental periodontal treatments; such findings, along with the lack of multiple teeth, should arouse the suspicion of LCH in children.

**Figure 2:**
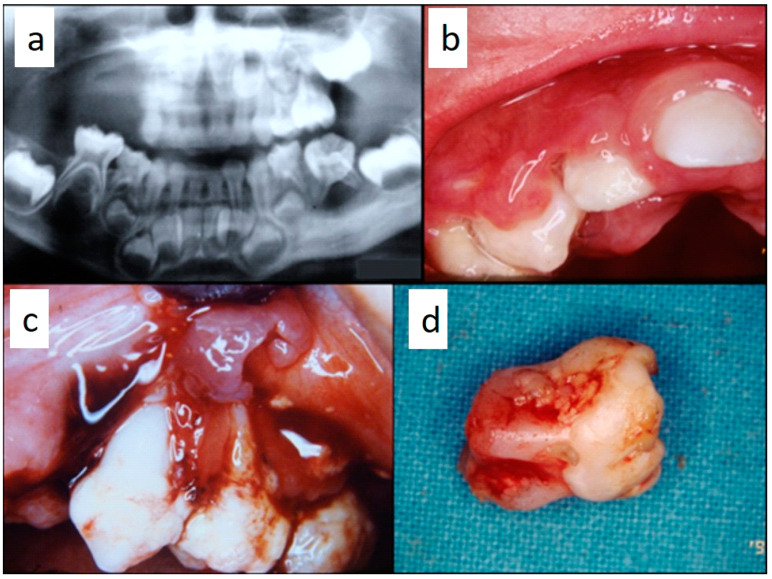
(**a**–**d**): Primary periodontal manifestations of LCH in a 5-year-old male: multiple osteolytic lesions in the mandible and in the maxilla (**a**) associated with gingival overgrowth and inflammation (**b**) and to painful ulceration of the gingiva with radicular exposure, bone loss (**c**) and tooth mobility with incomplete radicular formation due to the dental follicle involvement (**d**).

**Figure 3:**
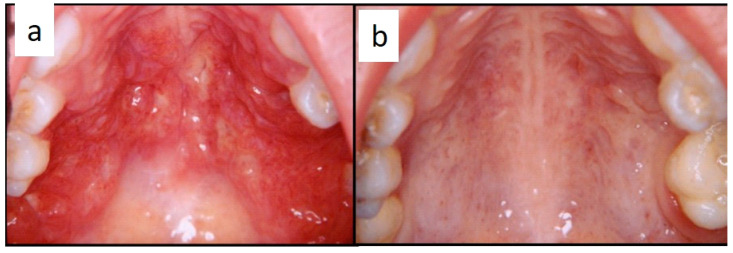
(**a**,**b**): Diffuse infiltration of the palatal mucosa with strawberry appearance as the first manifestation of LCH (**a**); complete regression after medical therapy (**b**).

**Figure 4:**
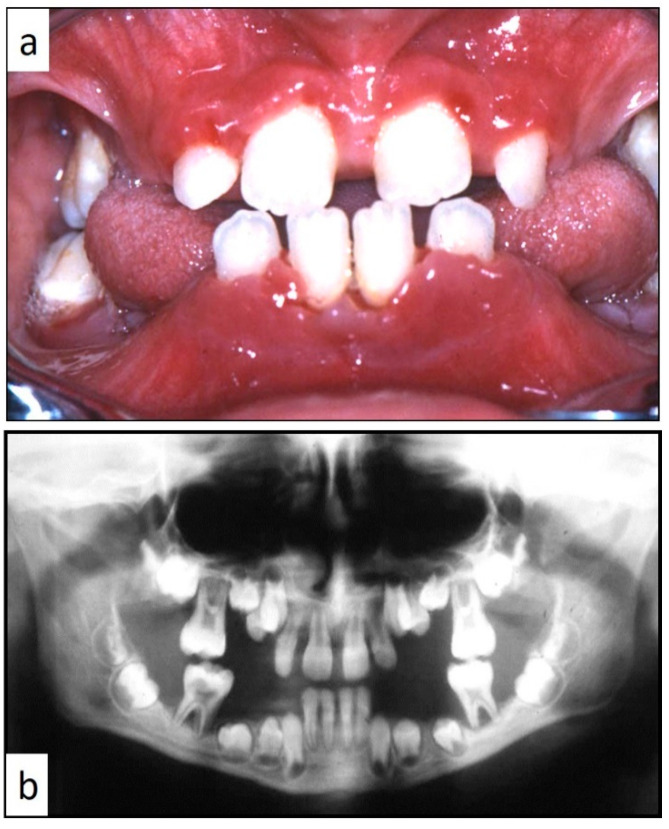
(**a**,**b**): Involvement of the entire periodontium, gingiva (**a**) and alveolar bone (**b**), of the mandible and maxilla causing premature loss of deciduous teeth; impressive bone resorption and following difficulties in permanent teeth development; the patient was a 11-year-old male, receiving treatment for gingival inflammation and teeth mobility for several months, thus causing a delayed diagnosis of LCH.

**Figure 5:**
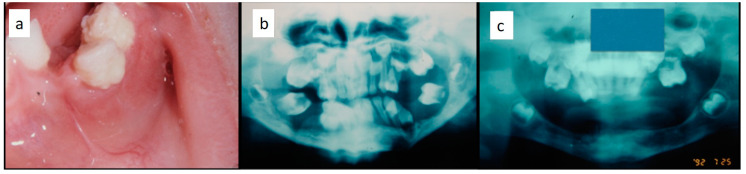
(**a**–**c**): Diffuse periodontal lesions in the mandible in a 5 year-old female patient causing teeth mobility with pathological fracture and dislocation on the left side (**a**,**b**); panoramic radiograph after medical and functional treatment (**c**).

**Figure 6:**
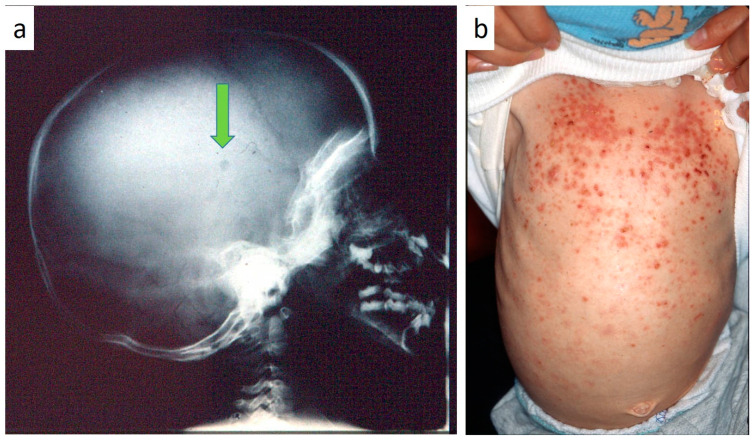
(**a**,**b**): Skull radiogram showing a small osteolytic lesion of the parietal bone (**a**); cutaneous rush occurring in children affected by LCH.

**Figure 7:**
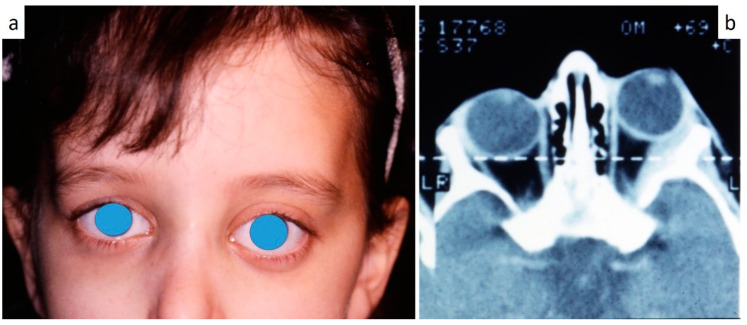
(**a**,**b**): Exophthalmia (**a**) in LCH patient as confirmed by CT scan (**b**).

Nine patients were affected by single eosinophilic granuloma of the mandible and two of the maxilla (0–10 A; 10–18 C). Inflamed, hyperplastic, painful and often ulcerated gingival lesions were common findings in 22 cases (0–10 A; 10–18 B) (Figure 1). They were associated with deciduous teeth mobility and/or dislocation with gingival probing and periodontal bone loss in 18 cases, and to premature teeth loss with dislocation of the permanent teeth follicles and bone lesions (single or multiple) in nine cases (0–10 B; 10–18 A) (Figure 2a–d). Focal gingival swelling (often with superficial ulceration) occurred in patients with single lesion of the jaws (five in the maxilla and three in the mandible), all appearing as radiolucency with well-defined borders but, however, with rapid bone erosion (0–10 A; 10–18 C). Single lesions of the palatal mucosa were detected as the first sign in six patients (0–10 A; 10–18 B), generally appearing as reddish or strawberry gingivitis with periodontal involvement and/or complete diffusion to the palate (0–10 C; 10–18 B) (Figure 3a,b). Nine patients showed diffuse lesion of the mandible with a progressive resorption of the alveolar bone with the characteristic “floating teeth” appearance (0–10 B; 10–18 A); in six cases, localized lesions of the maxilla were synchronously detectable (0–10 C; 10–18 B) (Figure 4a,b). In addition, patients with periodontal involvement had in common a delayed diagnosis of several months as they received treatments for gingival/periodontal diseases but were unresponsive to each one. The resorption of dental roots has never been observed on either deciduous or permanent teeth. Paresthesia, due to compression of the inferior alveolar nerve, was observed in only three cases, while in six instances a pathological fracture of the mandible occurred (0–10 A; 10–18 C) (Figure 5a–c), both resulting in expansion of an osteolytic lesion. Submental, submandibular and lateral-cervical lymph nodes involvement was associated with oral lesions in 12 cases (0–10 B; 10–18 B). Adjunctive radiological examination revealed lesions of the skull in 14 patients and, respectively, two of the parietal bone (Figure 6a), two of the temporal bone, one of the zygomatic bone, and three of the orbit (0–10 B; 10–18 C). Otitis (media or externa) was referred or detected in four instances, cutaneous rush in nine (0–10 B; 10–18 C) (Figure 6b), exophthalmia in two (Figure 7a,b), while data about the contextual presence or subsequent onset of insipidus diabetes were available only in eight cases (0–10 B; 10–18 C).

Laboratory tests, skeletal radiography, Ultrasound (US) or CT of abdomen, MRI or CT of the head were the basic investigations for general staging after the histological diagnosis of LCH by oral samples; further specific investigations were performed when necessary for other organ evaluation, according to the previous clinical and instrumental results.

As for therapy, the small single or multiple lesions of the jaws were surgically removed with contextual bone curettage to collect adequate samples for histological examination at the time of diagnosis. Chemotherapy with vinblastine alone or associated with corticosteroids were the principal treatment in almost 80% of cases; more than 50% of patients received corticosteroids, while only three patients received radiotherapy too. Recurrence of the disease was seen in eight patients after therapy. The overall mortality accounts for less than 9% (four of 45 cases), while data about follow-up are very interesting as 20 cases have been monitored for more than 15 years. These were those patients affected by localized jaws lesions or involving deciduous teeth or with widespread periodontal involvement, who experienced the removal of all (or almost all) teeth as heavily compromised and therefore received prosthetic rehabilitation.

**Figure 8:**
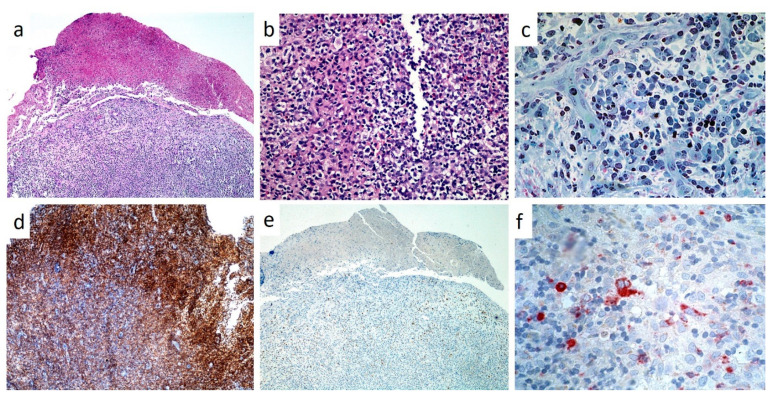
(**a**–**f**): Low (**a**, H and E, X4) and high power magnification (**b**, H and E, X10) of LCH of the gingiva, showing a partially ulcerated epithelium with abundant underlining infiltration of large cell with lobulated, indented nuclei and faintly eosinophilic cytoplasm intermixed with numerous eosinophils; toluidine blue staining on resin section (X20) showing large histiocytic cells with pale cytoplasm and eosinophils(**c**); strong immunostaining for CD1A (**d**) and S-100 (**e**), mild/moderate for CD68 (**f**).

In all cases, histopathological analysis of surgical samples from oral lesions revealed the presence of characteristic histiocyte-like cells that, in some fields, presented multi-lobulated nuclei and prominent nucleoli (Figure 8a,b). This predominant cell population was intermixed with a variable number of eosinophils and neutrophils and with chronic inflammatory infiltrate (Figure 8c); hemorrhagic exudate was occasionally observed. As for immunohistochemistry, all neoplasms stained positively for CD1a (Figure 8d), CD207 (Langerin) and S100 (Figure 8e); inconstant was the stain for CD34 and CD68 (approximately 60%) (Figure 8f).

## 4. Discussion

Although rare, LCH is well known and was first described in 1893, when Hand reported on the death of a 3-year-old child with exophthalmia, hepatosplenomegaly and polyuria, and subsequently Schüller and Christian reported pediatric cases with bone defects, exophthalmia and diabetes insipidus [7,14,15]. Such clinical similarities defined the Hand–Schüller–Christian disease. About three decades later, the Letterer–Siwe disease was defined by the distinct report of Letterer and Siwe on cases of entirely extra-skeletal acute non-leukemoid reactions of the reticuloendothelial system [16,17,18]. From 1940 onward, the first reports of eosinophilic granulomas of the skeletal system were described, starting with the case of Lichtenstein and Jaffe, and are all available in the current PubMed and/or Google Scholar databases [19]. Only in 1953, the common origin of the three apparently different diseases was recognized by Lichtenstein who proposed the term Histiocytosis X for the cytoplasmic inclusions, known as X bodies, he found in histiocytes [18,19,20]. The term of LCH was adopted by the scientific community in 1985 following the studies of Birbeck who recognized that the same X bodies were also present as inclusions in the Langerhans cell of the dermis [18,20].

The histological features of LCH have been well described and defined throughout the years. Electron microscopy was used in the past to detect X bodies and so as to confirm the diagnosis, but is nowadays no longer necessary [2,3]. The diagnosis of LCH is determined by conventional histology along with the immunohistochemical staining for CD1a and Langerin (CD207); the latter is the most recent introduced monoclonal antibody for LCH diagnosis, as highly specific and sensitive protein needed for formation of the Birbeck–Broadbent granules [1,2,3,5].

Nevertheless, the true etiopathogenesis of LCH remains still unclear. Leaving aside alleged theories of the past about a possible bacterial, viral, metabolic or traumatic etiology, LCH has been recently defined as an inflammatory neoplastic process caused by somatic mutations in bone marrow progenitor cells with contextual neoplastic and immunogenic qualities [21,22,23,24,25]. In fact, LCH lesions classically show highly inflammatory features but the real contribution of inflammation to the clinical manifestation is still undefined [22]. In addition, despite lesions having several features of malignancy, LCH cells usually shows a benign morphology and a low rate of mitosis, even comparable to that of normal epidermal Langerhans cells. Furthermore, the advances in sequencing technology allowed the identification of a recurrent somatic mutation of BRAF (most frequently BRAF V600E), a central kinase of the RAS/RAF/MEK pathway, resulting in a high activation in LCH cells of the downstream extracellular signal-regulated kinase and mitogen-activated protein kinase [22,25]. Further investigations on mutation assessments and the following therapeutic implications in the clinical risk stratification will surely play a key role in LCH patient management.

Despite all this, what really matters for clinicians in approaching suspicious LCH patients, especially pediatric ones, is the rarity, the non-specific clinical manifestations, and the mandatory need of adequate samples for histological examination, which overall, makes diagnosis always challenging and in most cases delayed [2,7,14]. Early diagnosis from primary oral and head/neck lesions in absence of adjunctive systemic signs and symptoms is certainly more complicated, especially when LCH lesions are single and involve the gingival tissues of a child, thus mimicking both inflammatory and neoplastic diseases. It may be less complicated to achieve the diagnosis by recognition of a single intraosseous lesion as its radiological appearance along with age (childhood), localization (anterior maxilla or mandible), onset and proliferation time (rapid) are quite similar. The same is for those cases showing multiple bone lesions (also of both jaws) with or without gingival enlargement/inflammation or teeth dislocation/loss and for patients with diffuse and severe periodontal involvement (with floating teeth) too [7,14,16,20].

Data from literature, along with the treatment guidelines of the Histiocyte Society, agree with the importance of the individual staging and classification of the disease, RO and special sites, to improve responsiveness to medical treatments and consequent survival rate [2,6,7,8]. The definition of RO^+/−^ and special sites made by the Histiocyte Society is surely important for the choice of treatment. While RO includes the hematopoietic system, spleen, liver, and special sites are craniofacial bones, eyes, ears, central nervous system and oral involvement too [8].

The overall differentiation by two age ranges (0–10-year-olds and 10–18-year-olds) for each lesion and the relative level of association frequency between clinical signs and disease (A = range 70–100%; B = range 40–70%; C = range 0–40%) we used in the current study, clearly highlights the differences of clinical presentation by age. Of note, the more or less aggressive (localized or generalized) periodontal involvement, with or without deciduous or permanent teeth loss and often leading to the pathological fracture of the mandible, mostly occurred within the first 10 years of life among our patients. The same goes for both the unifocal osteolysis and palatal mucosa involvement, although their clinical outcome is certainly more favorable and less disabling. Lymph nodes involvement, instead, shows no age-related differences among our cases.

In diagnosing LCH in childhood, clinicians must take into account several factors. First, the high clinical variability of oral lesions as follows: exclusive involvement of soft or even hard tissues; single or multiple bone lesions of one or both jaw or involving an entire jaw; oral mucosa infiltration with or without bone erosion, often mimicking pseudo-inflammatory or neoplastic lesions. Secondly, the non-specificity of the most frequent head/neck lesions such as lymph nodes enlargement or lateral neck masses, osteolytic lesions of the skull and especially of the temporal bone, overall characterized by a high rate of misdiagnosis [2,3,7,12,14,16,17,18,20].

In conclusion, LCH is a rare disease frequently diagnosed in advanced stages, especially in children. Its clinical presentation is highly variable, the clinical onset can be ambiguous, thus further contributing to the frequent diagnostic delay. Early diagnosis of LCH, especially in children, is achievable by identification of early clinical manifestations and their histological examination, which is mandatory for the onset of an effective medical treatment for the disseminated form and may lead to improved prognosis in all clinical forms of LCH.

For such reason, it may be suggestable that lateral neck enlargement, as well as swellings of the posterior mandible, with or without pain and not-related to dental infection, showing or not a clinical–radiological pattern of periodontal disease, should always raise the suspicion of undiagnosed systemic disease in childhood cases with oro-facial involvement. In such cases, any doubtful tissue needing removal (peri-radicular or periodontal inflammatory tissues, samples from bone curettage, etc.) must be adequately collected and mandatorily sent out for histological examination. This makes the role of the pediatric dentist or general dentist decisive for the early diagnosis of also rare diseases in pediatric age with possible primary or secondary head and neck localization, whether they be infectious or inflammatory diseases, or autoimmune or hematological disorders, including LCH.

## Figures and Tables

**Table 1:** Association frequency (range value) * of early/primary Dento/Oro/Facial clinical–radiological manifestations of LCH among our pediatric patients.

Sites	Oro-Facial Manifestations	Association Frequency—Age Range (0–10)	Association Frequency—Age Range (10–18)
**Gingiva–Periodontium**	Gingival Overgrowth	**A**	**B**
	Gingival Inflammation	**A**	**B**
	Gingival Ulceration	**A**	**B**
	Periodontal Bone Loss/Pocket >6 mm	**B**	**A**
	Radicular Exposure	**B**	**A**
	Teeth Mobility	**B**	**A**
	Premature Teeth Loss	**B**	**A**
	Deciduous Teeth Dislocation	**A**	**B**
**Oral Mucosa**	Localized Palatal Lesion	**A**	**B**
	Widespread Palatal Lesion (≥ 3 × 3 mm)	**C**	**B**
	Other Sites	**C**	**C**
**Jawbone**	Follicular Dislocation	**B**	**C**
	Pathological Fracture	**A**	**C**
	Maxillary Swelling	**A**	**C**
	Unifocal Osteolysis	**A**	**C**
	Multifocal Osteolysis	**C**	**B**
**Skull**		**B**	**C**
**Head and Neck Lymph Nodes**		**B**	**B**
**Other (Cutaneous Rash, Otitis, Exophthalmia)**		**B**	**C**
**Insipidus Diabetes**		**B**	**C**

* Corresponding values between association frequency and percentage range: A = 70–100%; B = 40–70%; C = 0–40%.

**Table 2:** Immunohistochemical panel used for differential diagnosis between JXG, LCH and HLH.

Histiocytic Proliferation Disease	Factor XIIIa	CD68	CD163	Fascin	CD14	CD1a	S100
JXG	+	+	+	+	+	−	−
LCH	−	+	−	−	−	+	+
HLH	−	+	+	−	+	−	+

Abbreviations: JXG: Juvenile Xanthogranuloma; LCH: Langherans Cell histiocytosis; HLH: Hemophagocytic Lymphohistiocytosis.
